# *Lactobacillus paracasei* KW3110 Prevents Inflammatory-Stress-Induced Mitochondrial Dysfunction in Mouse Macrophages

**DOI:** 10.3390/ijms23031443

**Published:** 2022-01-27

**Authors:** Takahiro Yamazaki, Sayuri Yamada, Konomi Ohshio, Miho Sugamata, Yuji Morita

**Affiliations:** KIRIN Central Research Institute, Kirin Holdings Co., Ltd., Fujisawa 251-8555, Kanagawa, Japan; Sayuri_Yamada@kirin.co.jp (S.Y.); Konomi_Ohshio@kirin.co.jp (K.O.); sgmtmh@gmail.com (M.S.); Yuji_Morita@kirin.co.jp (Y.M.)

**Keywords:** lactic acid bacteria, probiotics, inflammation, mitochondria, macrophage, interleukin-10

## Abstract

*Lactobacillus paracasei* KW3110 (KW3110) has anti-inflammatory effects, including the prevention of blue light exposure induced retinal inflammation and ageing-related chronic inflammation in mice. The mechanism involves the promotion of anti-inflammatory cytokine interleukin (IL)-10 production by KW3110, leading to reduced pro-inflammatory cytokine IL-1β production. Although various stress-induced mitochondrial damages are associated with excessive inflammatory responses, the effect of KW3110 on inflammatory-stress-induced mitochondrial damage remains unknown. In this study, we investigated the effect of KW3110 on inflammatory stress-induced mitochondrial damage using the murine macrophage-like cell line J774A.1. KW3110 treatment suppressed lipopolysaccharide (LPS)-induced mitochondrial dysfunction, including downregulation of membrane potential, induction of reactive oxygen species, and respiratory dysfunction. In addition, KW3110 prevented LPS-induced disruption of mitochondrial morphology including cristae structures. IL-10 treatment also ameliorated LPS-induced mitochondrial dysfunction and morphology disruption. These results suggest that KW3110 prevents LPS-induced mitochondrial dysfunction, potentially via promoting IL-10 production in mouse macrophages. We are the first to reveal a suppressive effect of lactic acid bacteria on mitochondrial morphology disruption in inflammatory-stressed macrophages. Our findings contribute to understanding inflammatory-stress-induced mitochondrial damage and developing food ingredients with preventive effects on mitochondrial-damage-derived inflammatory conditions.

## 1. Introduction

Animals have developed immune systems as a principal strategy to fight off endogenous or exogenous antigens and other stresses. To eliminate infectious factors and damaged cells and tissues and then initiate tissue repair, inflammation is an essential biological process in the immune response. A variety of daily stresses, including ageing, and bacterial and viral infections, are major causes of excessive or chronic inflammation [1,2,3,4]. An increasing number of studies has shown that stress-induced mitochondrial damage is associated with these inappropriate inflammatory responses; for example, cell injury induces the release of damage-associated molecular patterns from mitochondria, such as mitochondrial DNA, leading to inflammation related to human pathologies [5,6]. Ageing decreases the expression of genes that regulate mitochondrial quality control, resulting in chronic inflammation and ageing-associated pathologies [7]. These mitochondrial-damage-derived inflammatory responses result in various disorders or diseases that are affecting increasing numbers of people. Thus, strategies for preventing the conditions related to these stresses are required.

Some studies have been performed to investigate the immune-modulating functions of food materials to prevent inflammatory-stress-related disorders or diseases [8,9,10]. It is well-known that lactic acid bacteria (LAB) improve intestinal barrier function and the immune system and are consumed as sources of probiotics and paraprobiotics [11]. Additionally, some strains of LAB suppress inflammation-related disorders, including allergies and metabolic disorders. A strain of LAB administration has anti-allergic effects by directing the Th1/Th2 balance toward Th1 in the mouse allergy model [12]. A linoleic acid metabolite produced by intestinal LAB suppresses obesity-related metabolic disorders by activating brown adipose tissue through the gut–brain axis in obese and diabetic mice [13]. Although increasing numbers of reports have reported various functions of LAB, their detailed mechanisms have not yet been clearly elucidated.

Our previous reports have shown that a specific strain of LAB, *Lactobacillus paracasei* KW3110 (KW3110), has antiallergy and anti-inflammatory effects in mice and humans [12,14,15,16,17,18,19]. In addition, administration of whole bacteria of KW3110 prevented ageing-related chronic inflammation and blue light exposure induced retinal inflammation in mice [20,21]. The underlying mechanism of these anti-inflammatory effects of KW3110 are partially explained by the activation of anti-inflammatory M2 macrophages by KW3110, which promotes the production of anti-inflammatory cytokine interleukin (IL)-10; however, the effect of KW3110 on inflammatory-stress-induced mitochondrial damage remains unknown.

In this study, we investigated the effect of KW3110 on inflammatory-stress-induced mitochondrial damage, using lipopolysaccharide (LPS) as the inflammatory stress inducer and the murine macrophage-like cell line J774A.1. There is accumulated evidence that J774A.1 cells produce pro-inflammatory cytokines including IL-1β and tumor necrosis factor-α and anti-inflammatory cytokines including IL-10 in response to a variety of stimulations, and that mitochondrial DNA in the cells might be associated with pyroptosis induced by oxidized low-density lipoprotein, which is critical in atherosclerosis inflammation [22,23,24,25]. We performed several analyses of mitochondria in inflammatory-stress-induced J774A.1 cells and analyzed the effects of KW3110.

## 2. Results

### 2.1. Effect of KW3110 on Mitochondrial Dysfunction in Inflammatory-Stressed J774A.1 Cells

**Figure 1:**
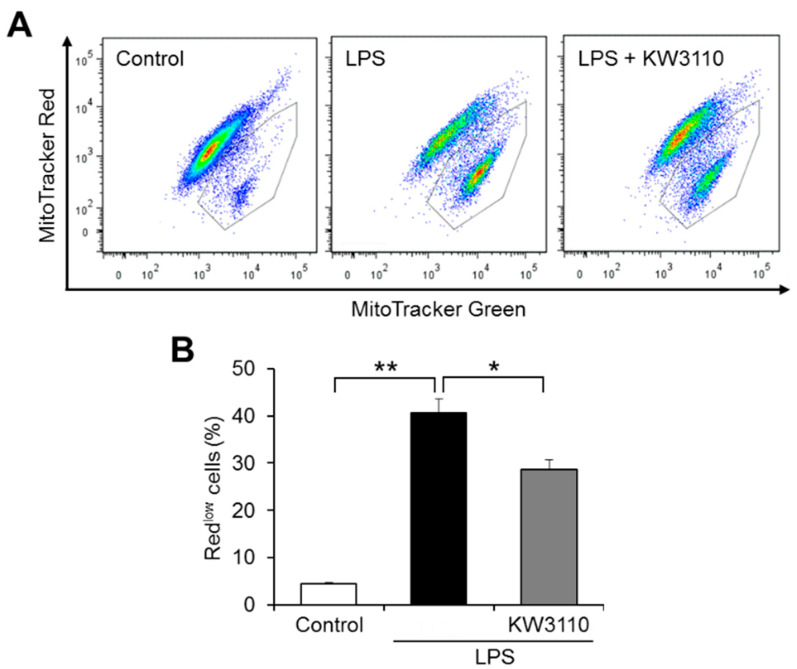
KW3110 treatment suppresses mitochondrial dysfunction in LPS-stimulated J774A.1 cells. J774A.1 cells were treated with KW3110 (1 μg/mL) for 24 h, followed by stimulation with LPS (0.1 μg/mL) for 24 h. Mitochondrial membrane potential was analyzed in cells labeled with MitoTracker Green (mitochondrial stain) and MitoTracker Red (mitochondrial stain dependent on membrane potential) (100 nM). (**A**) Representative flow cytometry plots of labeled cells. The plots surrounded by a gray line represent cells with lower MitoTracker Red intensity than MitoTracker Green intensity (Red^low^ cells). (**B**) The ratio of Red^low^ cells to total cells was calculated. Values represent means ± SEM. *n* = 3. Statistical differences were analyzed by ANOVA followed by Tukey’s test. * *p* < 0.05; ** *p* < 0.01.

To investigate whether KW3110 has a suppressive effect on inflammatory-stress-induced mitochondrial dysfunction in macrophages, we used a murine macrophage-like cell line J774A.1 [26]. Mitochondrial membrane potential, representing mitochondrial function, was evaluated by flow cytometry of cells labeled with fluorescent probes MitoTracker Green (mitochondrial stain) and MitoTracker Red (mitochondrial stain dependent on membrane potential) to stain total and active mitochondria, respectively. The ratio of cells with low mitochondrial membrane potential to total cells (Red^low^ cells) increased significantly in LPS-stimulated cells (inflammatory-stressed cells) compared to control cells (*p* < 0.001; Figure 1). The ratio in KW3110- and LPS-stimulated cells decreased significantly compared to that in LPS-stimulated cells (*p* = 0.015; Figure 1), suggesting that KW3110 suppresses mitochondrial dysfunction in inflammatory-stressed macrophages.

### 2.2. KW3110 Reduces Mitochondrial Reactive Oxygen Species (ROS) Production in Inflammatory-Stressed J774A.1 Cells

**Figure 2:**
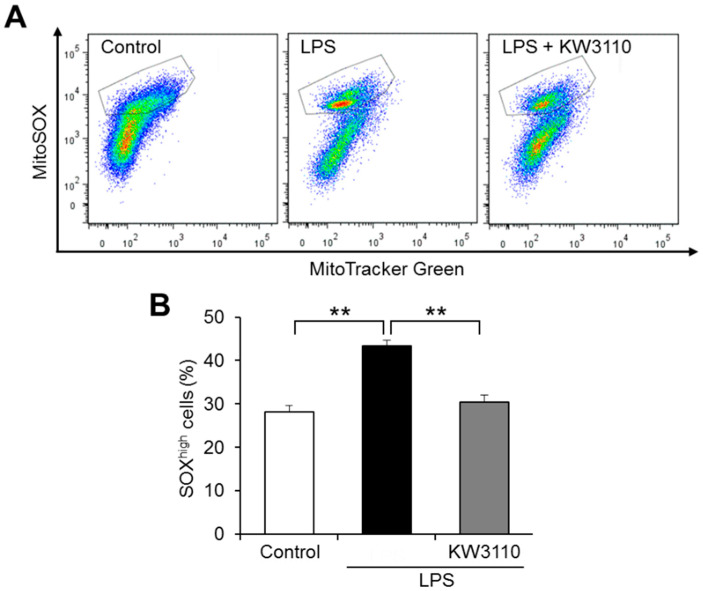
KW3110 treatment decreases mitochondrial ROS production in LPS-stimulated J774A.1 cells. Mitochondrial ROS production was analyzed in J774A.1 cells labeled with MitoTracker Green (100 nM) and MitoSOX (5 μM). Cells were treated with KW3110 (1 μg/mL) for 24 h, followed by stimulation with LPS (0.1 μg/mL) for 24 h. (**A**) Representative flow cytometry plots of labeled cells. The plots surrounded by gray lines represent cells with higher MitoSOX intensity than MitoTracker Green intensity (SOX^high^ cells). (**B**) The ratio of SOX^high^ cells to total cells was calculated. Values represent the means ± SEM. *n* = 3. Statistical differences were analyzed by ANOVA followed by Tukey’s test. ** *p* < 0.01.

Damaged mitochondria generate ROS, leading to oxidative stress and inflammatory response in cells [27]. We investigated whether KW3110 suppresses inflammatory-stressed ROS production in mitochondria by flow cytometry of J774A.1 cells labeled with MitoTracker Green (mitochondrial stain) and MitoSOX (ROS stain). While the ratio of cells with a high amount of ROS to total cells (SOX^high^ cells) increased significantly in LPS-stimulated cells compared to that in control cells (*p* < 0.001; Figure 2), the ratio in KW3110- and LPS-stimulated cells decreased significantly compared to that in LPS-stimulated cells (*p* = 0.002; Figure 2). These results suggest that KW3110 suppresses mitochondrial ROS generation in inflammatory-stressed macrophages.

### 2.3. Effect of KW3110 on Mitochondrial Respiratory Function in Inflammatory-Stressed J774A.1 Cells

**Figure 3:**
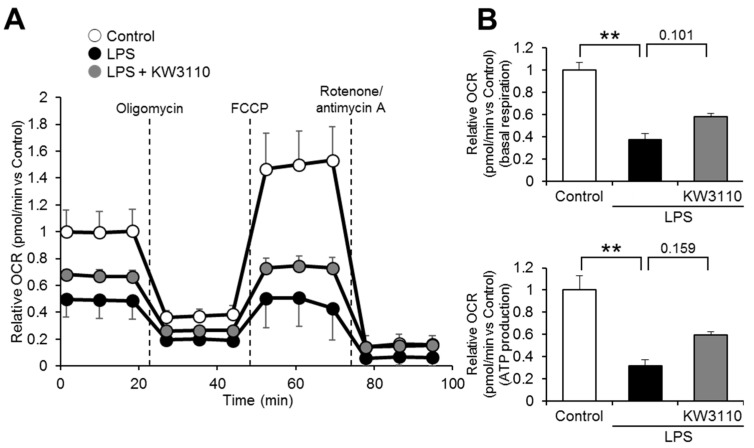
Effects of KW3110 treatment on mitochondrial respiratory function in LPS-stimulated J774A.1 cells. Mitochondrial respiration in cells was analyzed by an XFe24 Extracellular Flux Analyzer. (**A**) Real-time measurement of OCR was performed with an XF Cell Mito Stress Test, using oligomycin (1 μM), carbonyl cyanide 4-(trifluoromethoxy)phenylhydrazone (FCCP) (0.5 μM), and rotenone/antimycin A (0.5 μM), normalized to the control at 0 min. (**B**) Basal respiration was calculated by subtracting the average relative OCR after rotenone/antimycin A injection from that before oligomycin injection. Respiration for ATP production was calculated by subtracting the average relative OCR between oligomycin and FCCP injections from that before oligomycin injection, normalized to the control. Values represent means ± SEM. *n* = 5. Statistical differences were analyzed by analysis of variance (ANOVA) followed by Tukey’s test. ** *p* < 0.01.

One of the important roles of mitochondria is respiration, which is essential for generating energy for cell activities. Since mitochondrial respiratory function is downregulated in damaged mitochondria [28], we investigated whether KW3110 treatment restores mitochondrial respiratory function in inflammatory-stressed J774A.1 cells. The relative oxygen consumption rate (OCR) was lower in the LPS-stimulated cells than in the control cells at any time point (Figure 3A); the relative OCR in KW3110- and LPS-stimulated cells tended to be partially restored at all time points compared to that in LPS-stimulated cells, even though a significant difference was not detected (Figure 3A). While basal respiration significantly decreased in the LPS-stimulated cells compared to the control cells (*p* < 0.001; Figure 3B), the basal respiration in KW3110- and LPS-stimulated cells tended to be restored compared to that in LPS-stimulated cells (*p* = 0.101; Figure 3B). In addition, while adenosine 5′-triphosphate (ATP) production significantly decreased in LPS-stimulated cells compared to that in control cells (*p* < 0.001; Figure 3B), the ATP production in KW3110- and LPS-stimulated cells tended to be restored compared to that in LPS-stimulated cells (*p* = 0.159; Figure 3B). These results indicate that KW3110 might have the potential to suppress mitochondrial respiratory dysfunction in inflammatory-stressed macrophages.

### 2.4. IL-10 Suppresses Mitochondrial Dysfunction in Inflammatory-Stressed J774A.1 Cells

**Figure 4:**
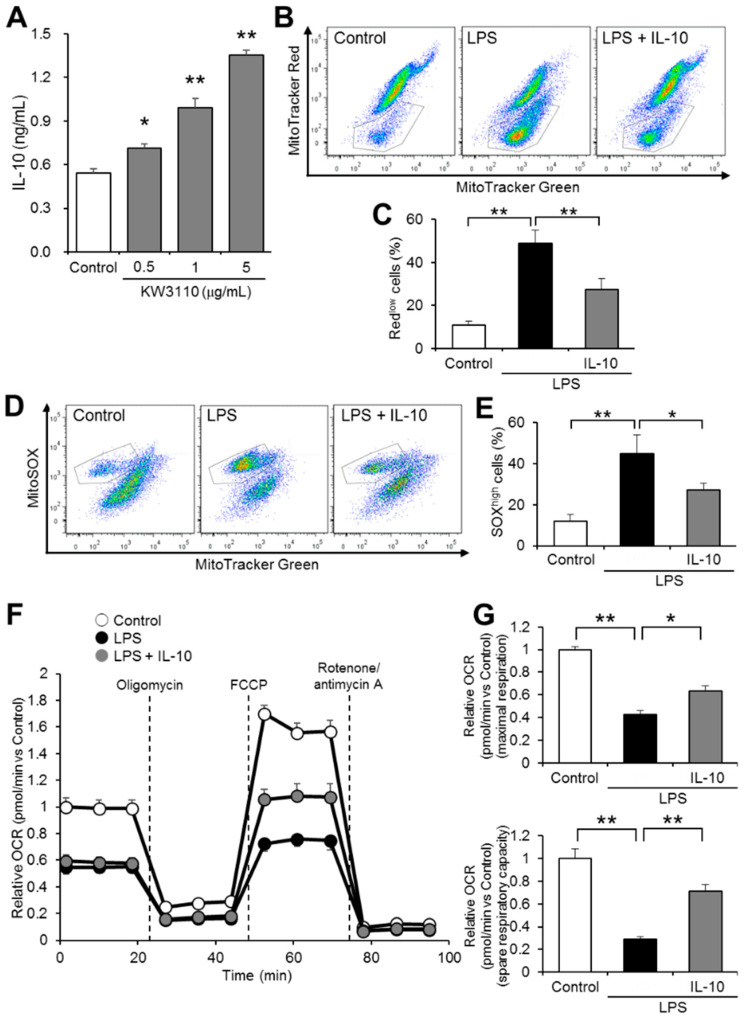
IL-10 treatment suppresses mitochondrial dysfunction in LPS-stimulated J774A.1 cells. (**A**) J774A.1 cells were treated with KW3110 (0.5, 1, or 5 μg/mL) for 24 h and IL-10 in the supernatant was measured by enzyme-linked immunosorbent assay (ELISA). (**B**–**E**) Mitochondrial membrane potential and mitochondrial ROS production were measured after cells were treated with IL-10 (1 μg/mL) and LPS (0.1 μg/mL) for 24 h. Representative flow cytometry plots of cells labeled with MitoTracker Green (100 nM) and MitoTracker Red (100 nM) are shown (**B**). The ratio of Red^low^ cells to total cells was calculated (**C**). Representative flow cytometry plots of cells labeled with MitoTracker Green (100 nM) and MitoSOX (5 μM) (**D**). The ratio of SOX^high^ cells to total cells was calculated (**E**). (**F**,**G**) Mitochondrial respiration in cells was analyzed by an XFe24 Extracellular Flux Analyzer. Real-time measurement of OCR was performed with an XF Cell Mito Stress Test, normalized to the control at 0 min (**F**). Maximal respiration was calculated by subtracting the average relative OCR before oligomycin injection from that between FCCP and rotenone/antimycin A injections. Spare respiratory capacity was calculated by subtracting the average relative OCR between FCCP and rotenone/antimycin A injections from that before oligomycin injection, normalized to the control. Values represent means ± SEM. *n* = 5. Statistical differences were analyzed by ANOVA followed by Dunnett’s or Tukey’s test. *, *p* < 0.05; **, *p* < 0.01.

Our previous studies have shown that KW3110 ameliorates the inflammatory responses of macrophages through promoting anti-inflammatory cytokine IL-10 production [17,18]. In this study, we investigated whether IL-10 suppresses inflammatory-stress-induced mitochondrial dysfunction in J774A.1 cells. We confirmed that KW3110 treatment significantly and dose-dependently increased IL-10 production in J774A.1 cells (0.5 μg/mL, *p* = 0.021; 1 μg/mL, *p* < 0.001; 5 μg/mL, *p* < 0.001; Figure 4A). While the ratio of Red^low^ cells increased significantly in LPS-stimulated cells compared to that of control cells (*p* < 0.001; Figure 4B,C), the ratio in IL-10- and LPS-stimulated cells decreased significantly compared to that in LPS-stimulated cells (*p* = 0.004; Figure 4B,C). In addition, while the ratio of cells with a high amount of ROS to total cells (SOX^high^ cells) increased significantly in LPS-stimulated cells compared to control cells (*p* = 0.001; Figure 4D,E), the ratio in IL-10- and LPS-stimulated cells decreased significantly when compared with LPS-stimulated cells (*p* = 0.026; Figure 4D,E). In considering the mitochondrial respiratory function, relative OCR was lower in the LPS-stimulated cells than in the control cells at any time point, and relative OCR in the IL-10- and LPS-stimulated cells was partially restored at all time points compared to that of the LPS-stimulated cells (Figure 4F). While maximal respiration significantly decreased in LPS-stimulated cells compared to control cells (*p* < 0.001; Figure 4G), the maximal respiration in IL-10- and LPS-stimulated cells was significantly restored compared to that in LPS-stimulated cells (*p* = 0.015; Figure 4G). In addition, while spare respiratory capacity significantly decreased in LPS-stimulated cells compared to the control cells (*p* < 0.001; Figure 4G), the spare respiratory capacity in IL-10- and LPS-stimulated cells was significantly restored compared to that in LPS-stimulated cells (*p* = 0.003; Figure 4G). These results suggest that IL-10 suppresses inflammatory-stressed mitochondrial dysfunction in macrophages.

### 2.5. Effect of KW3110 and IL-10 on Disruption of Mitochondrial Morphology in Inflammatory-Stressed J774A.1 Cells

**Figure 5:**
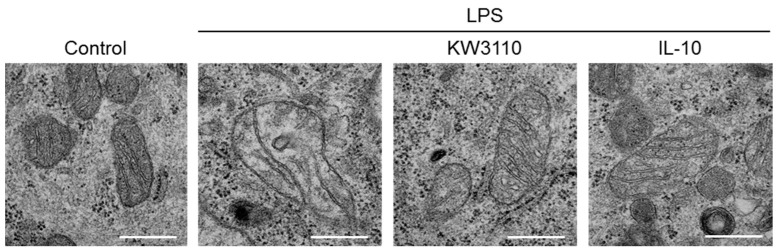
KW3110 or IL-10 treatment rescues disruption of mitochondrial structure in LPS-stimulated J774A.1 cells. J774A.1 cells were treated with KW3110 (1 μg/mL) or IL-10 (1 μg/mL) for 24 h, followed by stimulation with LPS (0.1 μg/mL) for 24 h. Mitochondrial images were obtained with transmission electron microscopy. Scale bar, 400 nm.

Mitochondrial respiratory dysfunction is associated with disruption of mitochondrial cristae structures because the cristae serve as the driving force for mitochondrial respiration by increasing the surface area of the inner membrane to enable greater amounts of mitochondrial-respiration-related proteins [29,30]. To investigate whether mitochondrial morphology is affected by experiencing inflammatory stress, we performed transmission electron microscope observation of J774A.1 cells. Under non-stimulating conditions, the mitochondria were elliptic, with normal cristae structures in the cells. Under LPS-stimulating conditions, the mitochondrial shape and cristae structures were disrupted (Figure 5). These disruptions were not observed in KW3110- and LPS-stimulated cells. In addition, the disruptions were not observed in IL-10- and LPS-stimulated cells. These results indicate that KW3110 and IL-10 suppress inflammatory-stressed disruption of mitochondrial morphology in macrophages.

## 3. Discussion

Our previous reports have shown that a specific strain of LAB, KW3110, has anti-inflammatory effects by activating anti-inflammatory M2 macrophages and promoting the production of IL-10; however, the effect on inflammatory-stress-induced mitochondrial damage was not elucidated. In this study, we demonstrated that KW3110 might have suppressed LPS-stimulation-induced mitochondrial dysfunction, including downregulation of membrane potential, induction of ROS production, and respiratory dysfunction, in mouse macrophages.

The downregulation of mitochondrial membrane potential and the induction of ROS production are caused by LPS stimulation to macrophages. It was reported that some plant-derived substances have antioxidant and anti-inflammatory activities, such as recovering mitochondrial membrane potential and suppressing ROS production, in LPS-stimulated macrophages [31,32]. Consistent with these reports, we found that KW3110 suppressed both the decrease in mitochondrial membrane potential and the increase in ROS production induced by LPS stimulation of J774A.1 cells (Figure 1 and Figure 2). In addition, KW3110 tended to ameliorate the decrease in mitochondrial respiratory capacity induced by LPS stimulation of J774A.1 cells (Figure 3). Since downregulation of mitochondrial membrane potential and respiratory dysfunction lead to the induction of ROS production [33], KW3110 is suggested to have a suppressive effect on a series of mitochondrial dysfunctions in inflammatory -tress-damaged macrophages. LPS triggers a variety of signaling pathways and many reports demonstrate the action of LPS on mouse macrophages [34,35]. For example, the nuclear transcription factor interferon regulatory factor-1 is a key factor contributing to LPS-induced oxidative stress and mitochondrial damage through accelerating ROS production, ATP depletion, superoxide dismutase consumption, and malondialdehyde accumulation [36]. Further investigation is required to understand how LPS influences mitochondrial functions in macrophages, and KW3110 has a preventive effect on LPS-induced mitochondrial dysfunction.

Using transmission electron microscope observation of J774A.1 cells, LPS stimulation of the cells was found to affect mitochondrial morphology (Figure 5); in particular, the cristae structures were disrupted. Since mitochondrial cristae increase the surface area of the inner membrane to enable greater amounts of mitochondrial-respiration-related proteins, which serve as the driving force for mitochondrial respiration, disruption of the cristae structures is associated with mitochondrial respiratory dysfunction [29,30]. In our study, KW3110 treatment of J774A.1 cells suppressed LPS-stimulated disruption of mitochondrial morphology, including cristae structures (Figure 5). This result indicates a novel mechanism: KW3110 prevents inflammatory-stress-induced mitochondrial respiratory dysfunction through suppressing the disruption of mitochondrial morphology, including that of cristae structures.

Inflammatory stimuli to cells induce mitochondrial dysfunction, leading to ROS overproduction and excessive inflammation, which damages mitochondria [37]. Previously, we showed that KW3110 activated anti-inflammatory M2 macrophages and enhanced IL-10 production, leading to suppress LPS-induced production of pro-inflammatory cytokines in macrophages. In this study, KW3110 treatment of J774A.1 cells induced IL-10 production in a dose-dependent manner (Figure 4A). IL-10 treatment of the cells suppressed LPS-stimulation-induced mitochondrial dysfunction, including downregulation of membrane potential, induction of ROS production, respiratory dysfunction, and disruption of mitochondrial morphology, as well as KW3110 treatment (Figure 4 and Figure 5). It was reported that IL-10 plays a critical role in the immune response and suppression of mitochondrial dysfunction, characterized by downregulation of membrane potential and induction of ROS production [38], which supports our findings. Although the detailed molecular mechanism of how KW3110 affects LPS-induced mitochondrial stresses must be studied in the future, these results suggest that KW3110 might have the potential to prevent inflammatory-stress-induced mitochondrial dysfunction in mouse macrophages through the promotion of IL-10 production.

In conclusion, our results indicate that KW3110 might have the potential to ameliorate LPS-stimulation-induced mitochondrial dysfunction, including downregulation of membrane potential, induction of ROS production, and respiratory dysfunction, in mouse macrophages. We are the first to reveal a preventive effect of LAB on mitochondrial morphology disruption in inflammatory-stressed macrophages. Our findings will help in constructing future therapeutic or preventive strategies for mitochondrial-damage-derived inflammatory conditions using food ingredients.

## 4. Materials and Methods

### 4.1. Materials

Whole bacteria of heat-killed KW3110 were prepared as described previously [16]. Ultrapure LPS from *E. coli* 0111:B4 strain was purchased from Invivogen (San Diego, CA, USA). MitoTracker Green FM (mitochondrial stain), MitoTracker Red CMXRos (mitochondrial stain dependent on membrane potential), and MitoSOX (mitochondrial ROS detector) were obtained from Invitrogen (Carlsbad, CA, USA). Recombinant mouse IL-10 protein was obtained from R&D Systems (Minneapolis, MN, USA).

### 4.2. Cell Culture

The J774A.1 cell line is derived from ascites obtained from an adult female mouse with reticulum cell sarcoma [26]. The cell line was purchased from ATCC (Manassas, VA, USA), and was maintained in Dulbecco’s modified Eagle medium (DMEM) with 10% fetal bovine serum, 100 U/mL penicillin, and 100 μg/mL streptomycin (Gibco, Grand Island, NY, USA) at 37 °C in 5% CO_2_ and air humidity.

### 4.3. Mitochondrial Membrane Potential and ROS Analyses by Flow Cytometry 

J774A.1 cells were grown overnight in 24-well plates (2.5 × 10^5^ cells/well). Cells were washed with phosphate-buffered saline (PBS) and exposed to 1 μg/mL KW3110 in DMEM for 24 h, followed by treatment with 1 μg/mL IL-10 and/or 0.1 μg/mL LPS for 24 h at 37 °C. After, cells were incubated at 37 °C for 15 min in FluoroBrite DMEM (Gibco, Waltham, MA, USA) with 100 nM MitoTracker Green FM, 100 nM MitoTracker Red CMXRos, or 5 μM MitoSOX. Cells were washed with the medium and collected into Eppendorf tubes. Flow cytometry analysis was performed on a FACS Canto II and analyzed using FlowJo software (BD Biosciences, San Jose, CA, USA). Mitochondrial membrane potential was analyzed in cells labeled with MitoTracker Green and MitoTracker Red, and the ratio of cells with lower MitoTracker Red intensity than MitoTracker Green intensity (Red^low^ cells) to total cells was calculated. Mitochondrial ROS production was analyzed in cells labeled with MitoTracker Green and MitoSOX and the ratio of cells with higher MitoSOX intensity than MitoTracker Green intensity (SOX^high^ cells) to total cells was calculated. While cells were treated with 10 μg/mL LPS for 4 h followed by treatment with ATP for 2 h aiming to prime and activate IL-1β in our previous study [17,19], in this study, we used a lower concentration of LPS alone to induce mitochondrial damage in accordance with the experimental condition of another group’s study [38,39]. Additionally, the concentration of KW3110 was lower in this study than that used in our previous study because the LPS concentration used in this study was low and KW3110 treatment (5 μg/mL and lower) with J774A.1 cells significantly induced IL-10 production (Figure 4A).

### 4.4. Mitochondrial Respiration Analysis

Mitochondrial respiration analysis was performed with an XFe24 Extracellular Flux Analyzer and its assay components (Agilent Technologies, Santa Clara, CA, USA). J774A.1 cells were grown overnight in 24-well XF plates (1.5 × 10^4^ cells/well). Cells were washed with PBS and exposed to 1 μg/mL KW3110 in DMEM for 24 h, followed by treatment with 1 μg/mL IL-10 and/or 0.1 μg/mL LPS for 24 h at 37 °C. Prior to starting the assay, cells were washed twice and incubated in XF base medium supplemented with 10 mM glucose, 1 mM sodium pyruvate, and 2 mM glutamine for 60 min at 37 °C without CO_2_ regulation. An XF Cell Mito Stress Test was performed by injecting 1 μM oligomycin (ATP synthase inhibitor), 0.5 μM FCCP (oxidative phosphorylation uncoupler), and 0.5 μM rotenone/antimycin A (mitochondrial complex I and III inhibitors) during the assay. OCR was measured in real time. Basal respiration was calculated by subtracting the average relative OCR after rotenone/antimycin A injection from that before oligomycin injection. Respiration for ATP production was calculated by subtracting the average relative OCR between oligomycin and FCCP injections from that before oligomycin injection. Maximal respiration was calculated by subtracting the average relative OCR before oligomycin injection from that between FCCP and rotenone/antimycin A injections. Spare respiratory capacity was calculated by subtracting the average relative OCR between FCCP and rotenone/antimycin A injections from that before oligomycin injection.

### 4.5. Determination of Cytokine Production

For measurement of mouse IL-10 level, cells were seeded and incubated overnight in 24-well plates (J774A.1 cells, 5 × 10^5^ cells/well) and washed with PBS, followed by treatment with several doses of KW3110 for 24 h at 37 °C. The supernatants were collected and centrifuged at 5000 rpm for 2 min. Mouse IL-10 levels were measured using a commercial ELISA kit (BD Biosciences, San Jose, CA, USA), according to manufacturer’s instructions.

### 4.6. Transmission Electron Microscopy

J774A.1 cells were grown overnight on 35 mm dishes (5 × 10^5^ cells). Cells were washed with PBS and exposed to 1 μg/mL KW3110 or 1 μg/mL IL-10 in DMEM for 24 h, followed by treatment with 0.1 μg/mL LPS for 24 h at 37 °C. Cells were fixed with 2% paraformaldehyde and 2% glutaraldehyde (GA) in 0.1 M phosphate buffer (PB) at 37 °C, and were fixed with 2% GA in 0.1 M PB at 4 °C overnight. They were then washed three times with 0.1 M PB and postfixed with 2% osmium tetroxide in 0.1 M PB at 4 °C for 1 h. The samples were dehydrated in 50% and 70% ethanol for 5 min each at 4 °C, 90% ethanol for 5 min at room temperature (RT), and three changes of 100% ethanol for 5 min at RT. They were transferred to Quetol-812 (Nisshin EM, Tokyo, Japan) and polymerized at 60 °C for 48 h. The polymerized samples were ultra-thin sectioned at 70 nm with a diamond knife using an Ultracut UCT (Leica, Vienna, Austria) and mounted on copper grids. They were stained with 2% uranyl acetate at RT for 15 min, and were washed with distilled water followed by being secondary-staining with lead stain solution (Sigma, St. Louis, MO, USA) at RT for 3 min. The grids were observed by JEM-1400Plus (JEOL, Tokyo, Japan) at an acceleration voltage of 100 kV. Digital images were taken with EM-14830RUBY2 (JEOL, Tokyo, Japan).

### 4.7. Statistical Analysis

Values are reported as means ± SEM. Statistical differences were analyzed by ANOVA followed by Dunnett’s or Tukey’s test. *p*-values < 0.05 were considered statistically significant.

## Data Availability

The data presented in this study are available in the article.

## Abbreviations

LAB	lactic acid bacteria
KW3110	*Lactobacillus paracasei* KW3110
IL	interleukin
LPS	lipopolysaccharide
ROS	reactive oxygen species
DMEM	Dulbecco’s modified Eagle medium
PBS	phosphate-buffered saline
OCR	oxygen consumption rate
ELISA	enzyme-linked immunosorbent assay
GA	glutaraldehyde
PB	phosphate buffer
RT	room temperature
ANOVA	analysis of variance
ATP	adenosine 5′-triphosphate
